# Pretreatment neutrophil-to-lymphocyte ratio predicts the benefit of gastric cancer patients with systemic therapy

**DOI:** 10.18632/aging.203256

**Published:** 2021-07-10

**Authors:** Songtao Du, Zhenhao Fang, Lin Ye, Huiyan Sun, Guangtong Deng, Wei Wu, Furong Zeng

**Affiliations:** 1 Department of Oncology and Geratic Surgery, Xiangya Hospital, Central South University, Changsha, China; 2 National Clinical Research Center for Geriatric Disorders, Xiangya Hospital, Central South University, Changsha, China; 3 Department of Colorectal Surgical Oncology, Harbin Medical University Cancer Hospital, Harbin, China; 4 Department of Dermatology, Hunan Engineering Research Center of Skin Health and Disease, Hunan Key Laboratory of Skin Cancer and Psoriasis, Xiangya Hospital, Central South University, Changsha, Hunan, China

**Keywords:** neutrophil-to-lymphocyte, gastric cancer, treatment, meta-analysis

## Abstract

Pretreatment neutrophil-to-lymphocyte ratio (NLR) has been reported to be associated with the prognosis of inoperable gastric cancer patients with systemic therapy. However, no consensus on the association has been reached. In this study, we mainly evaluated whether pretreatment NLR predicted the benefit of inoperable gastric cancer patients with systemic therapy, including chemotherapy, targeted therapy and immunotherapy. PubMed, Embase and Cochrane Library databases were systematically searched from inception up to September 16th, 2020. A total of 36 studies including 8614 patients were involved in the meta-analysis. Pooled data revealed that high pretreatment NLR was significantly associated with poor outcomes of OS (HR = 1.78, 95% CI = [1.59, 1.99]) and PFS (HR = 1.63, 95% CI = [1.39, 1.91]) in gastric cancer. Subgroup analyses stratified by country, study type, case load, analysis of HR, cutoff of pretreatment NLR, or treatment types arrived at the same conclusion. Pooled data based on different effect models and sensitivity analyses did not change the conclusion. Overall, high pretreatment NLR predicts the poor prognosis of inoperable gastric cancer patients with systemic therapy. Measurement of pretreatment NLR will assist clinicians with patient counseling and clinical treatment guiding accordingly.

## INTRODUCTION

Gastric cancer is one of the most common malignant tumors, ranking the third highest mortality worldwide [1]. Although its morbidity is declining in most countries, the increase of incidence in the under-50 population could reverse the overall decline in gastric cancer [2, 3]. Moreover, though advances in diagnosis and surgical treatment have reduced mortality for early-stage gastric cancer [4], many patients were often initially diagnosed at advanced stages [5], which highlights the importance of effective systemic therapy.

Systemic therapy consists of chemotherapy, targeted therapy, and immunotherapy [6]. Chemotherapy is a relatively traditional therapy mainly based on fluoropyrimidine and platinum agents. It is the first-line treatment for metastatic gastric patients with human epidermal growth factor receptor-2 (HER-2)-negative expression in accordance with the latest international guidelines [7–9]. While, targeted therapy with trastuzumab is recommended to treat gastric cancer patients with HER2 overexpression [10, 11]. Usually, those patients are treated with trastuzumab as well as the first-line chemotherapy [7, 8, 12]. Immunotherapy has emerged as a powerful treatment for chemo-refractory gastric cancer [2]. Chemotherapy combined with immunotherapy might achieve better therapeutic efficacy compared with chemotherapy alone [2]. Overall, systemic therapy has revolutionized the treatment and improved the prognosis of patients with inoperable gastric cancer. Therefore, identifying novel biomarkers is of great significance to predict the outcome of inoperable gastric cancer patients with systemic therapy.

Neutrophil-to-lymphocyte ratio (NLR) is well-known as a systemic inflammation biomarker, which could be accessed from blood routine easily. Previous meta-analyses showed that NLR was a prognostic biomarker of gastric cancer, especially after gastrectomy [13–15]. Moreover, increasing studies demonstrated the correlation between pretreatment NLR and the gastric cancer prognosis after systemic therapy [16–51]. However, there is a lack of meta-analysis to comprehensively evaluate the association between pretreatment NLR and the outcomes of systemic therapy for inoperable gastric cancer.

Therefore, from the above and with the introduction of systemic therapy for advanced inoperable gastric cancer patients, it is timely to systematically review the association between pretreatment NLR and therapeutic efficacy of gastric cancer patients with systemic therapy, including chemotherapy, targeted therapy and immunotherapy.

## RESULTS

### Literature search and studies characteristics

**Figure 1:**
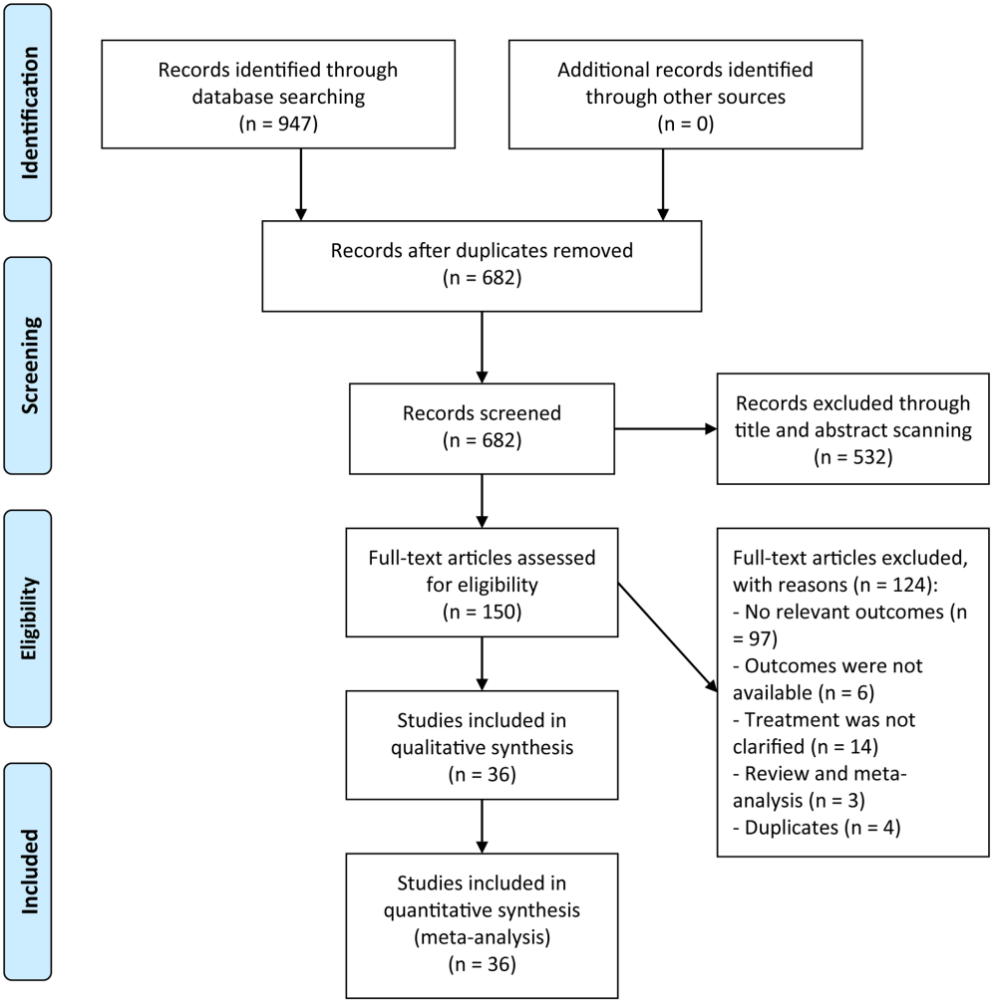
A flowchart of the study selection.

A flow chart of study selection was presented in Figure 1. The initial searching retrieved 947 relevant studies. After the removal of duplicated studies, 682 studies remained, of which 532 studies were ruled out after a scanning of the titles and abstracts. Full-test article evaluation for eligibility were implemented in 150 studies, among which 124 studies were removed owing to 97 studies with no relevant outcomes, six with unavailable outcomes, 14 without clarifying treatment types, three being review or meta-analyses, and four duplicates. Eventually, a total of 36 studies were of eligibility and enrolled into the meta-analysis [16–51].

Table 1 summarized the characteristics of eligible studies published between 2007 and 2020, and from four different regions including Japan, China, Korea, or Europe. Among them, there were five multi-center studies and 30 single-center studies. The cutoffs of NLR were not consistent in these studies, ten of which used three as the cutoff of high versus low pretreatment NLR. In terms of systemic treatment, 20 studies assessed the prognostic significance of pretreatment NLR in chemotherapy, 13 studies in chemo/targeted therapy, and 3 studies in immunotherapy. All studies reported on overall survival (OS) and 17 studies reported on progression-free survival (PFS). The Newcastle-Ottawa Scale (NOS) was adopted to evaluate the methodological quality of eligible studies for observational studies [52]. All studies were identified as high quality with stars above six on the basis of quality assessment. The response of each individual study to NOS was exhibited in Table 2.

**Table 1:** Characteristics of eligible studies.

**First author**	**Year**	**Country**	**Study type**	**Cases**	**Age (years)**	**Sex (male %)**	**Cutoff**	**Treatment**	**Variables**	**NOS scores**	**References**
Yamanaka	2007	Japan	Multi-center	1220	–	869 (71.2%)	2.5	Chemotherapy	OS^*^	8	[16]
Jeong	2012	Korea	Single-center	104	52.8 ± 10.7	69 (66.3%)	3	Chemotherapy	OS^*^, PFS	8	[17]
Lee	2013	Korea	Single-center	174	–	114 (65.5%)	3	Chemotherapy	OS^*^, PFS^*^	8	[18]
Cho	2014	Korea	Single-center	268	55.4 ± 12.5	175 (65.3 %)	3.06	Chemotherapy	OS^*^, PFS^*^	9	[19]
Dogan	2015	Turkey	Single-center	109	53.9 ± 9.1	80 (73.4%)	2.5	Chemotherapy	OS, PFS	7	[20]
Liu	2015	China	Single-center	135	61.1 ± 12.1	79 (58.5%)	4	Chemotherapy	OS	7	[21]
Wang	2015	China	Single-center	120	66.9 ± 9.75	75 (62.5%)	4.62	Chemotherapy	OS, PFS	7	[22]
Zhang	2015	China	Multi-center	99	–	76 (76.8%)	4.558	Chemotherapy	OS	7	[23]
Hsieh	2016	China	Single-center	256	59.7 ± 10.5	176 (68.8%)	3	Chemotherapy	OS^*^	9	[24]
Musri	2016	Turkey	Single-center	143	59.0 ± 12.0	103 (72.0%)	3.34	Chemotherapy	OS, PFS	7	[25]
Wang	2016	China	Single-center	310	57.7 ± 9.6	213 (68.7%)	median	Chemotherapy	OS^*^	9	[26]
Giampieri	2017	Italy	Single-center	103	–	71 (68.9%)	0.4	Chemotherapy	OS, PFS	7	[27]
Gonda	2017	Japan	Single-center	100	65.2 ± 9.0	56 (56.0%)	3	Chemotherapy	OS^*^	9	[28]
Manikhas	2017	Russia	Single-center	32	60.5	–	3	Chemotherapy	OS^*^	8	[29]
Marshall	2017	Japan	Single-center	143	–	–	3.11	Chemo/targeted therapy	OS^*^	7	[30]
Ock	2017	Korea	Single-center	745	59.8 ± 11.0	534 (71.7 %)	2.42	Chemo/targeted therapy	OS^*^	9	[31]
Huang	2018	China	Single-center	136	55.1 ± 10.9	82 (60.3%)	3.04	Chemotherapy	PFS^*^	9	[32]
Hwang	2018	Korea	Single-center	73	61.7 ± 14.0	61 (83.6%)	3	Chemo/targeted therapy	OS^*^, PFS^*^	9	[33]
Kim	2018	Korea	Single-center	502	57.7 ± 10.1	300 (59.8%)	3	Chemotherapy	OS^*^, PFS^*^	9	[34]
Kondoh	2018	Japan	Single-center	50	65.2 ± 9.4	29 (58.0%)	3.5	Chemo/targeted therapy	OS^*^	9	[35]
Migita	2018	Japan	Single-center	177	67.6 ± 11.3	124 (70.1%)	2.2	Chemo/targeted therapy	OS^*^	9	[36]
Ogata	2018	Japan	Multi-center	26	64.3 ± 10.6	19 (73.1%)	5	Immunotherapy	OS, PFS	7	[37]
Ryu	2018	Korea	Multi-center	236	58.8 ± 10.0	185 (78.4%)	2.08	Chemotherapy	OS^*^, PFS^*^	9	[38]
Bozkurt	2019	Turkey	Single-center	194	58.7 ± 9.4	129 (66.5%)	2.6	Chemo/targeted therapy	OS^*^, PFS	8	[39]
Mitani	2019	Japan	Multi-center	112	61.4 ± 10.6	84 (75.0%)	3	Chemo/targeted therapy	OS	7	[40]
Murakami	2019	Japan	Single-center	92	–	73 (79.3%)	2.83	Chemo/targeted therapy	OS^*^	8	[41]
Namikawa	2019	Japan	Single-center	262	68.1 ± 12.4	171 (65.3%)	3.9	Chemo/targeted therapy	OS^*^	9	[42]
Sugimoto	2018	Japan	Single-center	141	71.9 ± 10.6	98 (69.5%)	4	Chemo/targeted therapy	OS^*^	9	[43]
Cipriano	2020	Portugal	Single-center	55	62.0 ± 9.2	43 (78.2%)	5	Chemo/targeted therapy	OS^*^	9	[44]
Kim	2020	Korea	Single-center	1156	57.3 ± 12.3	738 (63.8%)	3	Chemo/targeted therapy	OS^*^	9	[45]
Namikawa	2020	Japan	Single-center	21	70.2 ± 9.1	19 (65.5%)	2.5	Immunotherapy	OS, PFS	7	[46]
Ota	2020	Japan	Single-center	98	65.1 ± 10.2	68 (69.4%)	3	Immunotherapy	OS^*^, PFS	8	[47]
Shigeto	2020	Japan	Single-center	109	69.1 ± 5.9	85 (78.0%)	3.15	Chemo/targeted therapy	OS	7	[48]
Wang	2020	China	Single-center	466	59.8 ± 11.3	327 (70.2%)	2.8	Chemotherapy	OS^*^, PFS	8	[49]
Zhao	2020	China	Single-center	110	–	84 (76.4%)	2.48	Chemotherapy	OS^*^	8	[50]
Zhou	2020	China	Single-center	537	55.0 ± 9.5	321 (59.8%)	2.610	Chemotherapy	OS^*^, PFS^*^	9	[51]

^*^Variables are calculated by multivariable analysis. Abbreviations: OS, overall survival; PFS, progression-free survival; NOS, Newcastle-Ottawa Scale.

**Table 2:** Methodological quality of studies included in the meta-analysis based on Newcastle-Ottawa Scale.

**First author**	**Year**	**Selection**	**Comparison**	**Exposure/Outcome**	**Total score**	**References**
Yamanaka	2007	****	*	***	8	[16]
Jeong	2012	****	*	***	8	[17]
Lee	2013	****	*	***	8	[18]
Cho	2014	****	**	***	9	[19]
Dogan	2015	****	–	***	7	[20]
Liu	2015	****	–	***	7	[21]
Wang	2015	****	–	***	7	[22]
Zhang	2015	****	–	***	7	[23]
Hsieh	2016	****	**	***	9	[24]
Musri	2016	****	–	***	7	[25]
Wang	2016	****	**	***	9	[26]
Giampieri	2017	****	–	***	7	[27]
Gonda	2017	****	**	***	9	[28]
Manikhas	2017	****	*	***	8	[29]
Marshall	2017	****	–	***	7	[30]
Ock	2017	****	**	***	9	[31]
Huang	2018	****	**	***	9	[32]
Hwang	2018	****	**	***	9	[33]
Kim	2018	****	**	***	9	[34]
Kondoh	2018	****	**	***	9	[35]
Migita	2018	****	**	***	9	[36]
Ogata	2018	****	–	***	7	[37]
Ryu	2018	****	**	***	9	[38]
Bozkurt	2019	****	*	***	8	[39]
Mitani	2019	****	–	***	7	[40]
Murakami	2019	****	*	***	8	[41]
Namikawa	2019	****	**	***	9	[42]
Sugimoto	2018	****	**	***	9	[43]
Cipriano	2020	****	**	***	9	[44]
Kim	2020	****	**	***	9	[45]
Namikawa	2020	****	–	***	7	[46]
Ota	2020	****	*	***	8	[47]
Shigeto	2020	****	–	***	7	[48]
Wang	2020	****	*	***	8	[49]
Zhao	2020	****	*	***	8	[50]
Zhou	2020	****	**	***	9	[51]

### Correlation between pretreatment NLR and OS

**Figure 2:**
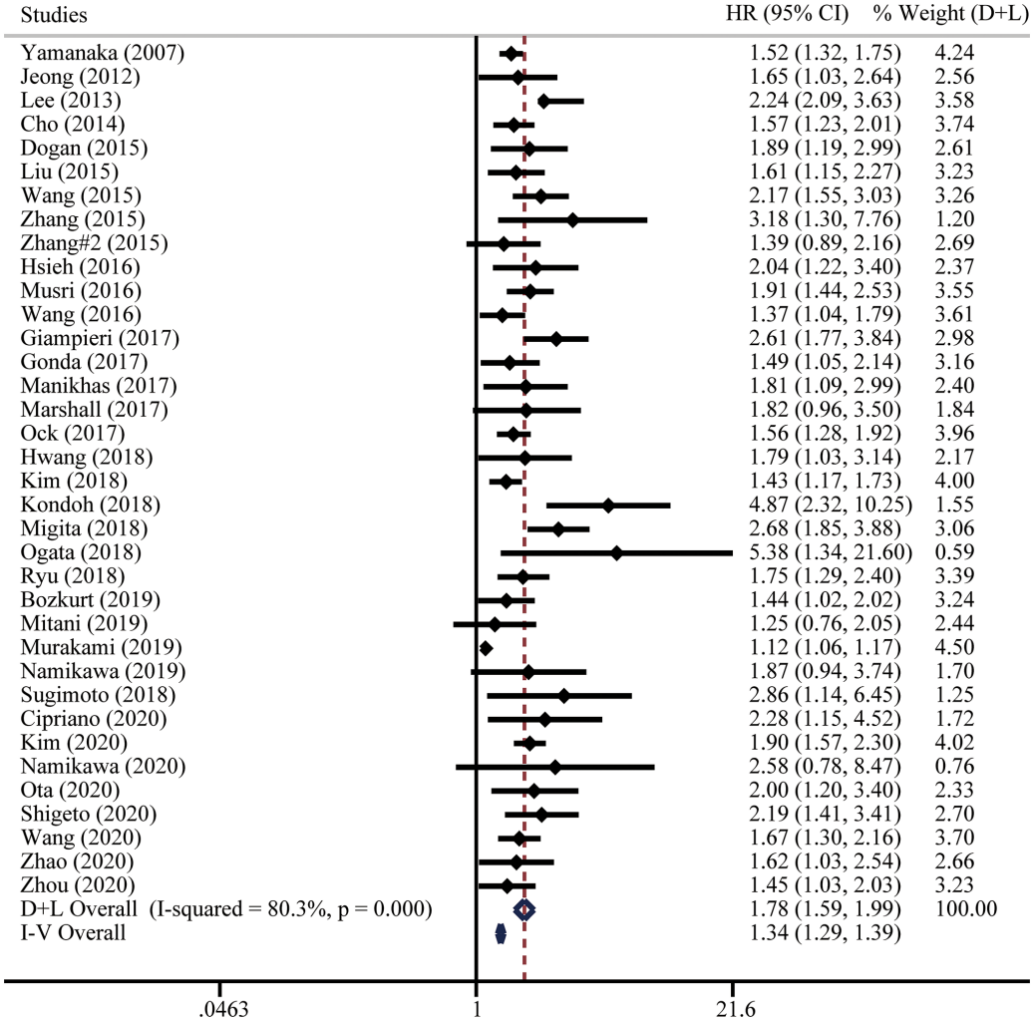
**Forest plot for the hazard ratios (HRs) of overall survival (OS) in gastric cancer patients with systemic therapy between low and high pretreatment NLR.** “D+L” means DerSimonian and Laird method. “I-V” means generic inverse variance method.

Thirty-six studies comprising of 8614 gastric cancer patients reported the association between pretreatment NLR and OS. With great heterogeneity (I^2^ = 80.3%, *P* < 0.001), we utilized random effect model to analyze the pooled hazard ratio (HRs) and results showed that higher pretreatment NLR was correlated with a poorer OS (HR = 1.78, 95% confidential interval (CI) = [1.59, 1.99]) (Figure 2). Analysis with fixed effect model showed a consistent conclusion (Figure 2). The conclusion also remained unchanged with sensitivity analysis (Supplementary Figure 1A).

**Figure 3:**
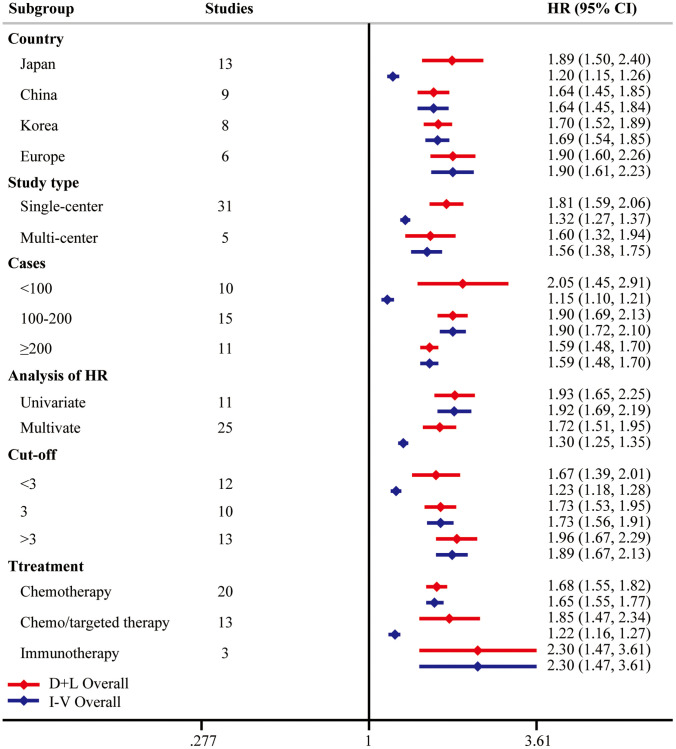
**Subgroup analysis of OS.** “D+L” means DerSimonian and Laird method. “I-V” means generic inverse variance method.

Considering the existence of heterogeneity, univariate meta-regression analysis was performed and indicated that case load and cutoff of pretreatment NLR could be the possible significant moderators (Table 3). Then we adopted subgroup analyses following these clinical parameters. Notably, higher pretreatment NLR was correlated with poorer OS from multi-center studies (HR = 1.60, 95% CI = [1.32, 1.94]) and multivariate analysis (HR = 1.72, 95% CI = [1.51, 1.95]). Moreover, subgroup analysis on the basis of the NLR cutoff values demonstrated that the prognostic value of pretreatment NLR consisted in all the NLR groups (<3 HR = 1.67, 95% CI = [1.39, 2.01]; = 3 HR = 1.73, 95% CI = [1.53, 1.95]; >3 HR = 1.96, 95% CI = [1.67, 2.29]). Subgroup analysis by treatment types suggested the same conclusions in chemotherapy (HR = 1.68, 95% CI = [1.55, 1.82]), chemo/targeted therapy (HR = 1.85, 95% CI = [1.47, 2.34]), and immunotherapy (HR = 2.30, 95% CI = [1.47, 3.61]). The results of subgroup analyses were summarized in Figure 3, highlighting that elevated pretreatment NLR was correlated with poor OS.

**Table 3:** Univariate meta regression of hazard ratios (HRs) of overall survival (OS) in inoperable gastric cancer patients with systemic therapy.

**Variables**	**β**	**95% LCI**	**95% UCI**	***P***
Country				
Japan	1.00	0.82	1.23	0.991
China	0.94	0.76	1.17	0.589
Korea	0.98	0.80	1.20	0.817
Europe	1.13	0.88	1.44	0.341
Study type	0.92	0.71	1.20	0.545
Cases				
<100	0.99	0.78	1.26	0.952
100–200	1.17	0.99	1.39	0.072
>200	0.87	0.72	1.05	0.141
Analysis of HR	0.86	0.70	1.06	0.153
Cut-off				
<3	0.90	0.75	1.08	0.235
=3	0.99	0.81	1.22	0.98
>3	1.20	0.98	1.47	0.08
Treatment				
Chemotherapy	0.96	0.79	1.16	0.665
Chemo/targeted therapy	1.00	0.82	1.22	0.994
Immunotherapy	1.39	0.80	2.40	0.234

Abbreviations: LCI, Lower confidence interval; UCI, Upper confidence interval.

For OS subset, the asymmetry of funnel plot indicated that there existed publication bias (Supplementary Figure 1B). Egger’s test was used for further validation (Supplementary Figure 1C). The Duval and Tweedie trim-and-fill method was then conducted and twelve studies were filled, without changing the conclusion in both fixed effect model (HR = 1.32, 95% CI = [1.27, 1.36]) and random effect model (HR = 1.58, 95% CI = [1.43, 1.74]).

### Correlation between pretreatment NLR and PFS

**Figure 4:**
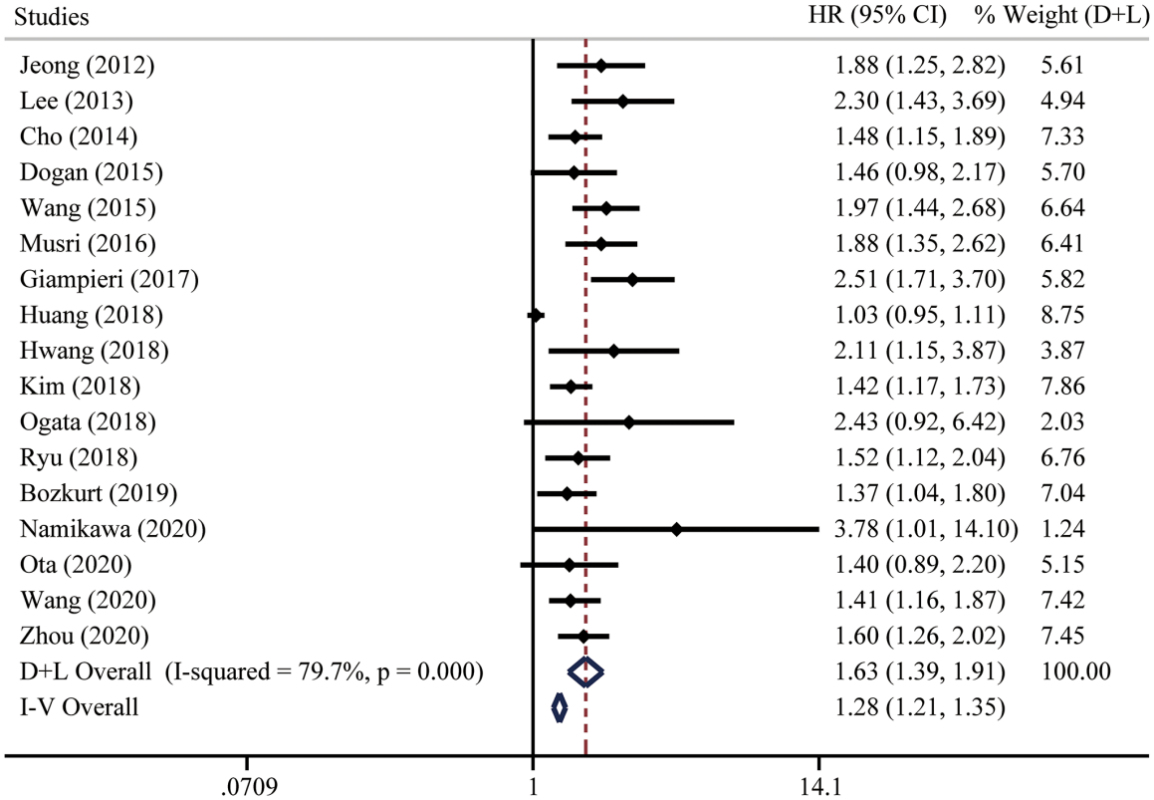
**Forest plot for the HRs of progression-free survival (PFS) in gastric cancer patients with systemic therapy between low and high pretreatment NLR.** “D+L” means DerSimonian and Laird method. “I-V” means generic inverse variance method.

Seventeen studies including 3318 patients were included to analyze the relationship between pretreatment NLR and PFS. Due to significant heterogeneity, we applied a random effect model and the results suggested that higher pretreatment NLR was related to inferior PFS (HR = 1.63, 95% CI = [1.39, 1.91]) (Figure 4). The fixed effect model (Figure 4) and sensitivity analysis (Supplementary Figure 2A) did not change the conclusion.

**Figure 5:**
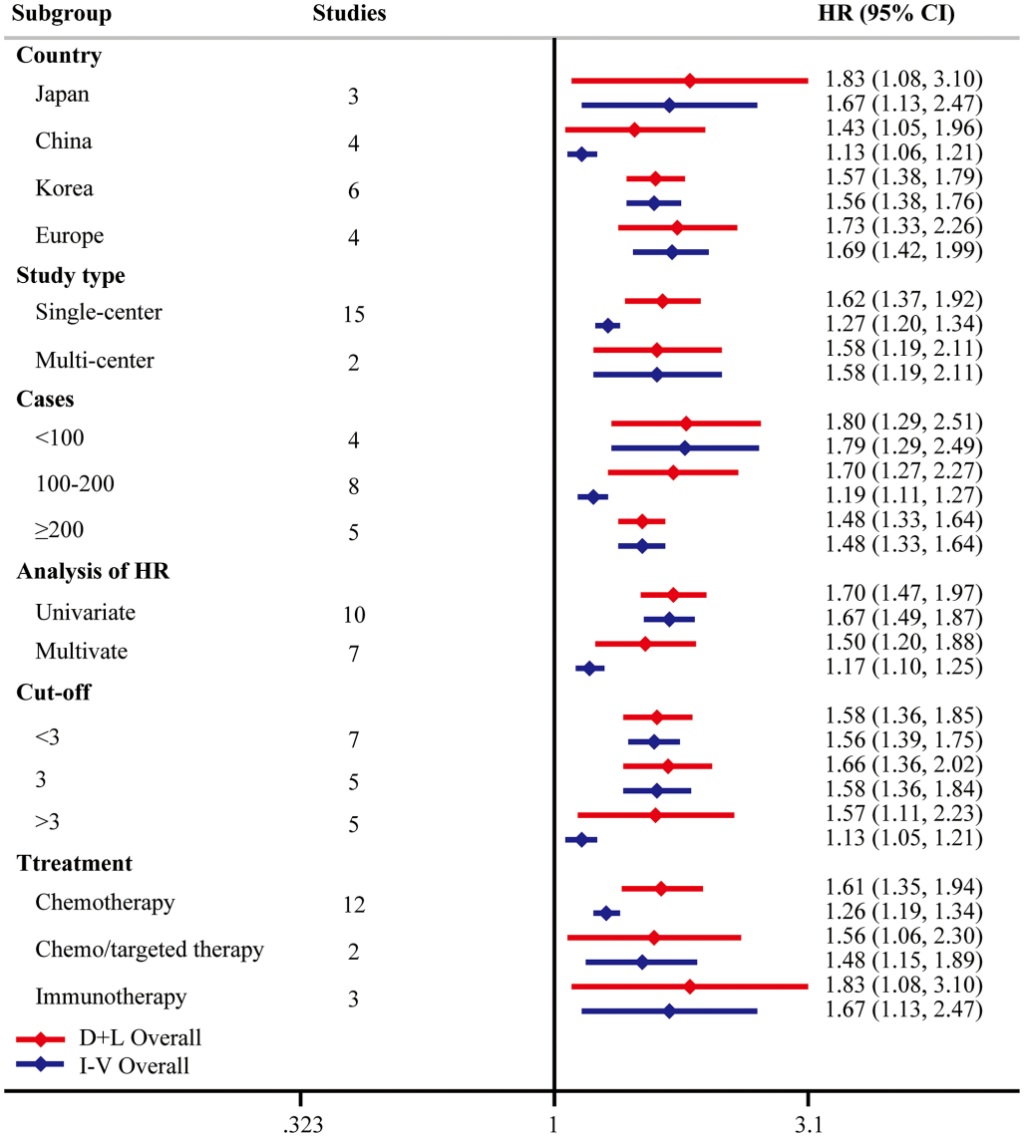
**Subgroup analysis of PFS.** “D+L” means DerSimonian and Laird method. “I-V” means generic inverse variance method.

To investigate the origin of heterogeneity, univariate meta-regression was performed. We did not find the possible significant moderator (Table 4). To validate the robustness of the results, subgroup analyses were performed based on country, study type, case load, analysis of HR, cutoff of pretreatment NLR, or treatment types. The conclusions were consistent in all the subgroup analyses (Figure 5). Noteworthily, stratified analysis demonstrated that higher pretreatment NLR was correlated with poorer OS from multi-center studies (HR = 1.58, 95% CI = [1.19, 2.11]) and multivariate analysis (HR = 1.50, 95% CI = [1.20, 1.88]). When three was determined as the cutoff of NLR, significant differences were found in all these subgroups (<3 HR = 1.58, 95% CI = [1.36, 1.85]; =3 HR = 1.66, 95% CI = [1.36, 2.02]; >3 HR = 1.57, 95% CI = [1.11, 2.23]). Subgroup analysis based on treatment types suggested that the prognostic significance of pretreatment NLR existed in all kinds of systemic therapy, including chemotherapy (HR = 1.61, 95% CI = [1.35, 1.94]), chemo/targeted therapy (HR = 1.56, 95% CI = [1.06, 2.30]), and immunotherapy (HR = 1.83, 95% CI = [1.08, 3.10]).

**Table 4:** Univariate meta regression of hazard ratios (HRs) of progression-free survival (PFS) in inoperable gastric cancer patients with systemic therapy.

**Variables**	**β**	**95% LCI**	**95% UCI**	***P***
Country				
Japan	1.13	0.65	1.96	0.647
China	0.83	0.62	1.10	0.176
Korea	1.06	0.79	1.43	0.668
Europe	1.11	0.80	1.54	0.497
Study type	1.10	0.73	1.67	0.618
Cases				
<100	1.20	0.76	1.91	0.411
100-200	1.05	0.79	1.40	0.719
>200	0.88	0.66	1.19	0.381
Analysis of HR	0.85	0.65	1.11	0.204
Cut-off				
<3	1.01	0.76	1.35	0.919
=3	1.09	0.79	1.51	0.561
>3	0.91	0.67	1.22	0.498
Treatment				
Chemotherapy	0.96	0.66	1.39	0.819
Chemo/targeted therapy	0.98	0.61	1.56	0.919
Immunotherapy	1.13	0.65	1.96	0.647

Abbreviations: LCI: Lower confidence interval; UCI: Upper confidence interval.

For PFS subset, the funnel plot was not symmetrical and five studies were over the pseudo 95% CI (pseudo 95% CI was defined as 95% CI assuming these included studies did not have heterogeneity [53]) (Supplementary Figure 2B). We then performed the Egger’s test, which detected the existence of publication bias (*P* < 0.001) (Supplementary Figure 2C). Twelve studies were filled after the Duval and Tweedie trim-and-fill method. The conclusion remained consistent in both fixed effect model (HR = 1.27, 95% CI = [1.20, 1.34]) and random effect model (HR = 1.55, 95% CI = [1.34, 1.80]).

## DISCUSSION

Gastric cancer has become a threat worldwide with over one million estimated new cases and about 784000 deaths globally annually [1]. Even worse, many cases were diagnosed at advanced stages and lost the chance of gastrectomy. Systemic therapy has been recommended to treat those patients with inoperable gastric cancer, but the prognostic biomarkers have not been well clarified.

Increasing studies demonstrated the association of systemic inflammation and the prognosis of gastric cancer with systemic therapy [54–57]. As the representative of systemic inflammation, NLR is easily calculated from regular blood tests. There were accumulating studies on the topic of the prognostic effect of pretreatment NLR on the gastric cancer patients with systemic therapy [16–51]. Therefore, an extensive analysis on the topic is essential to clarify the association.

In this study, a total of 36 studies including 8, 614 patients were finally enrolled through searching all the relevant articles. We found that higher pretreatment NLR was associated with an inferior OS and PFS. Considering the huge heterogeneity in these comparisons, univariate meta-regression analyses were conducted to investigate the origin of heterogeneity. Case load and cutoff of pretreatment NLR could be the possible significant moderators for OS. Moreover, six studies used the Engauge Digitizer software to estimate the univariate HRs, which were grouped into subgroup with univariate analysis. However, the conclusions were not changed by fixed effect model, sensitivity analyses and subgroup analyses, which highlights the prognostic value of pretreatment NLR in inoperable gastric cancer patients with systemic therapy.

Currently, quite a few meta-analyses with regard to the prognostic effect of NLR on gastric cancer were published. In 2015, Chen et al. performed a meta-analysis based on nine studies including 3709 gastric cancer patients, and suggested that higher pretreatment NLR was associated with poorer OS and PFS in gastric cancer patients undergoing resection and palliative chemotherapy [15]. However, this study did not describe the prognostic effect of pretreatment NLR on targeted therapy or immunotherapy. Moreover, Sun and his colleagues included 19 studies in the meta-analysis and validated Chen et al.’s conclusion [14]. Furthermore, Kim et al. comprehensively assessed the association between the OS of gastric cancer patients and NLR. They included 24 studies to analyze the pooled HRs of OS but did not report the prognostic effect of NLR on the prognosis of inoperable gastric cancer patients with systemic therapy [13]. Overall, these studies mainly focused on the gastric cancer patients with gastrectomy, while we concentrated on inoperable gastric cancer patients with systemic therapy. We highlighted the prognostic effect of pretreatment NLR not only on the gastric cancer patients with chemotherapy, but also on gastric cancer patients with chemo/targeted therapy and immunotherapy.

The mechanisms underlying the relationship between pretreatment NLR and the prognosis of inoperable gastric cancer patients with systemic therapy were poorly known, but many studies provided the potential mechanisms [13–15, 58]. In summary, most neutrophils promote the progression of tumors through inhibiting immune activity, while lymphocytes are regarded as the primary effector cells in the immunotherapy. NLR is calculated by circulating neutrophil to lymphocyte counts, which reflects a balance between the detrimental roles of neutrophilia and the beneficial roles of lymphocyte-mediated immunity [59]. Even so, more studies are still needed to investigate the underlying mechanism in the association. There are other predictive biomarkers with prognostic value in gastric cancer patients with systemic therapy. For example, a recent study showed that an immune checkpoint score system could be used for the evaluation of prognosis and the selection for adjuvant chemotherapy in gastric cancer [60]. Moreover, a deep learning computed tomography (CT) signature was developed to predict the prognosis and benefit from adjuvant chemotherapy in gastric cancer [61]. Many single biomarkers such as MTA1 [62], TFF3 [63], and CA72-4 [64] were also reported to be related to the prognosis of gastric cancer with systemic therapy. As a simple and feasible biomarker, NLR is easily obtained from the regular blood tests, which highlights its practicability in clinical practice.

Admittedly, some limitations existed within our meta-analysis. First, two eligible studies were meeting abstracts providing limited data and this could be improved by updating with the latest data. Second, considerable heterogeneity existed in the meta-analysis, though sensitivity analyses and subgroup analyses did not change the conclusion. Third, publication bias existed in both OS and PFS, though the Duval and Tweedie trim-and-fill method indicated the same trend of the results. Finally, NLR is a non-specific biomarker and could be affected by the concurrent disease, such as infections and drug therapy. Most of the studies did not include these descriptions.

In conclusion, as a simple, inexpensive and readily available biomarker, NLR could be used to predict the benefit of inoperable gastric cancer patients with systemic therapy. Measurement of this biomarker before treatment will assist clinicians with patient counseling and clinical treatment guiding accordingly.

## MATERIALS AND METHODS

### Search strategy

We implemented the meta-analysis according to the Preferred Reporting Items for Systematic Reviews and Meta-Analyses (PRISMA) statement [65, 66]. The databases of the PubMed, Embase and Cochrane libraries were retrieved from inception to September 16^th^, 2020. The search terms were indicated as below: “Stomach neoplasms” OR “gastric and (cancer or carcinoma? or adenocarcinoma? or neoplasm? or neoplasia)” OR “stomach adj3 (cancer or carcinoma? or adenocarcinoma? or neoplasm? or neoplasia)” AND (“Neutrophil-Lymphocyte ratio" OR "Neutrophil Lymphocyte ratio" OR "Neutrophil-to-Lymphocyte ratio" OR "Neutrophil to Lymphocyte ratio" OR "Neutrophil/Lymphocyte ratio" OR NLR). There was no limitation of language and study type. References lists of eligible articles and main reviews were explored manually to guarantee a thorough literature search. We have registered our systematic review in PROSPRO website (https://www.crd.york.ac.uk/PROSPERO/). The identifier of systematic review registration was PROSPERO CRD42021224114.

### Selection criteria

NLR was defined as absolute neutrophil counts divided by absolute lymphocyte counts. Studies eligible for inclusion should satisfy the following inclusion criteria: (1) the patients with metastatic or inoperable gastric cancer; (2) receiving systemic therapy, including chemotherapy, targeted therapy and immunotherapy; (3) accessible HR and their corresponding 95% CI for OS and PFS between high and low pretreatment NLR group; (4) nonrandomized studies with or without the use of randomized samples. Exclusion criteria were as follows: (1) studies receiving gastrectomy or not specifying therapy types; (2) studies including patients with other types of tumors without performing of subgroup analysis about gastric cancer; (3) duplicated studies with small sample size in the same institutes or hospitals; (4) studies with insufficient usable data; (5) review, case reports or meta-analyses.

### Data extraction and quality assessment

Two authors (FZ, ZF) autonomously selected eligible studies, and discordance was resolved by a third author (GD). The following information were collected from eligible studies: first authors, published year, country, type of study, case load, age, gender, cutoff of pretreatment NLR, treatment types, HR and their corresponding 95% CI for OS and PFS. HRs were extracted from multivariable analyses preferentially where available; otherwise, HRs were retrieved from univariate analyses. If studies did not report specified HRs, Engauge Digitizer software was adopted to digitize and estimate HRs from Kaplan-Meier curves between high and low NLR groups [67, 68]. Six studies in the meta-analysis used the Engauge Digitizer software to estimate the univariate HRs [20–23, 25, 48]. Newcastle-Ottawa Scale (NOS) was used for quality evaluation in three aspects: selection, comparability and outcome [52]. Studies with stars above six were regarded as high-quality.

### Statistical analysis

STATA software (Version 12.0; STATA Corporation) was applied for all the statistical analyses. HRs with their corresponding 95% CI were pooled to evaluate the survival values. Statistical heterogeneity was assessed with I^2^ and *P*-value. Considering the existence of heterogeneity in the comparisons, random effect model was preferentially performed for all the analyses. To ensure the robustness of the results, fixed effect model was also performed in all the analyses. Univariate meta-regression analysis was conducted to investigate the origin of heterogeneity. Moreover, sensitivity analysis was executed by omitting one study each time as previously described [69]. Subgroup analyses were used to test the consistency of the results based on country, study type, case load, analysis of HR, cutoff of pretreatment NLR, or treatment types. Funnel plots and Egger’s tests were performed to assess publication bias. Duval and Tweedie trim-and-fill method was used for the adjustment of the publication bias. *P* value less than 0.05 was regarded statistically significant.

## Supplementary Materials

Supplementary Figures
