# Supplementary material for: Pretreatment neutrophil-to-lymphocyte ratio predicts the benefit of gastric cancer patients with systemic therapy
Source: Aging (Albany NY). 2021 Jul 10;13(13):17638–54. doi: 10.18632/aging.203256 (PMC8312446; doi:10.18632/aging.203256)
Supplement: Supplementary Figures [file aging-13-203256-s001.pdf]

SUPPLEMENTARY FIGURES

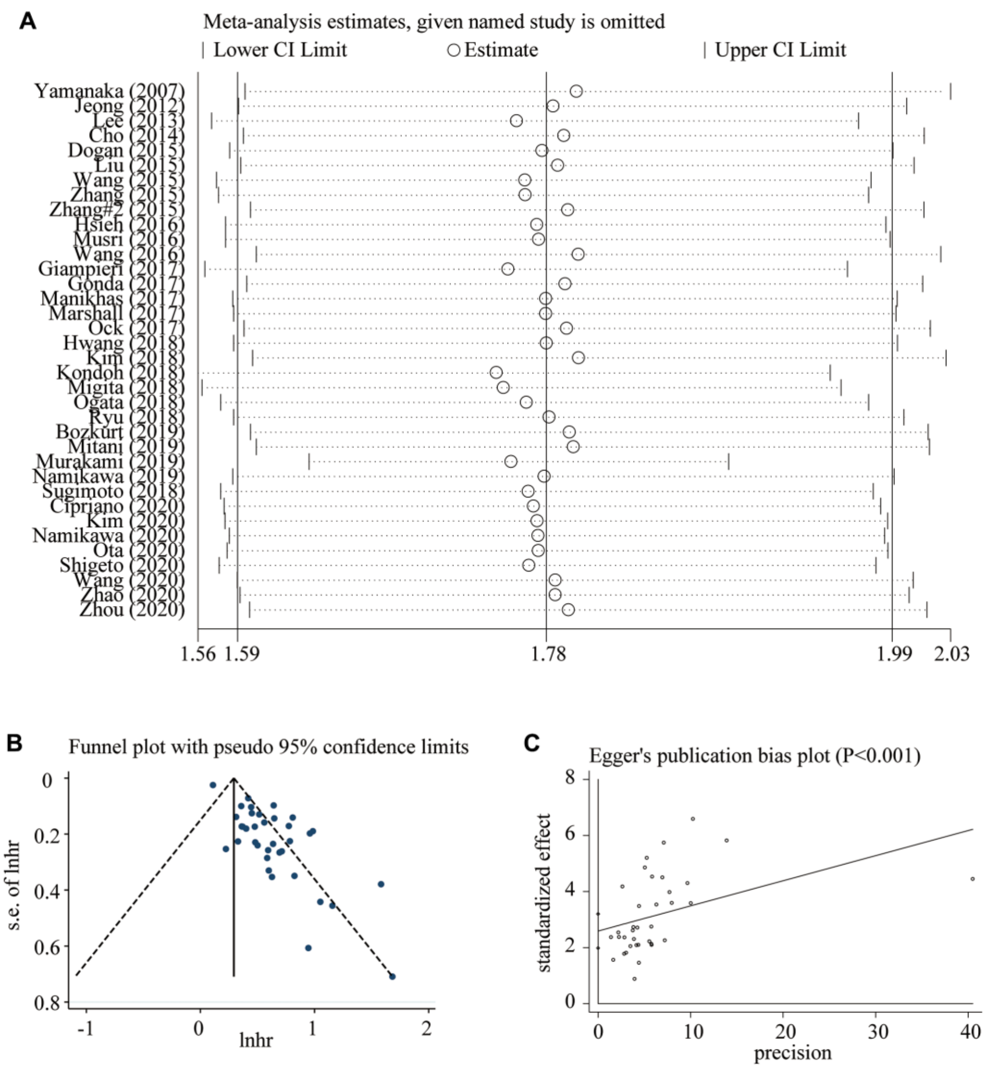

Supplementary Figure 1. Sensitivity analysis (**A**), Funnel plot (**B**) and Egger test (**C**) for the pooled HRs of OS in gastric cancer patients with systemic therapy between low and high pretreatment NLR.

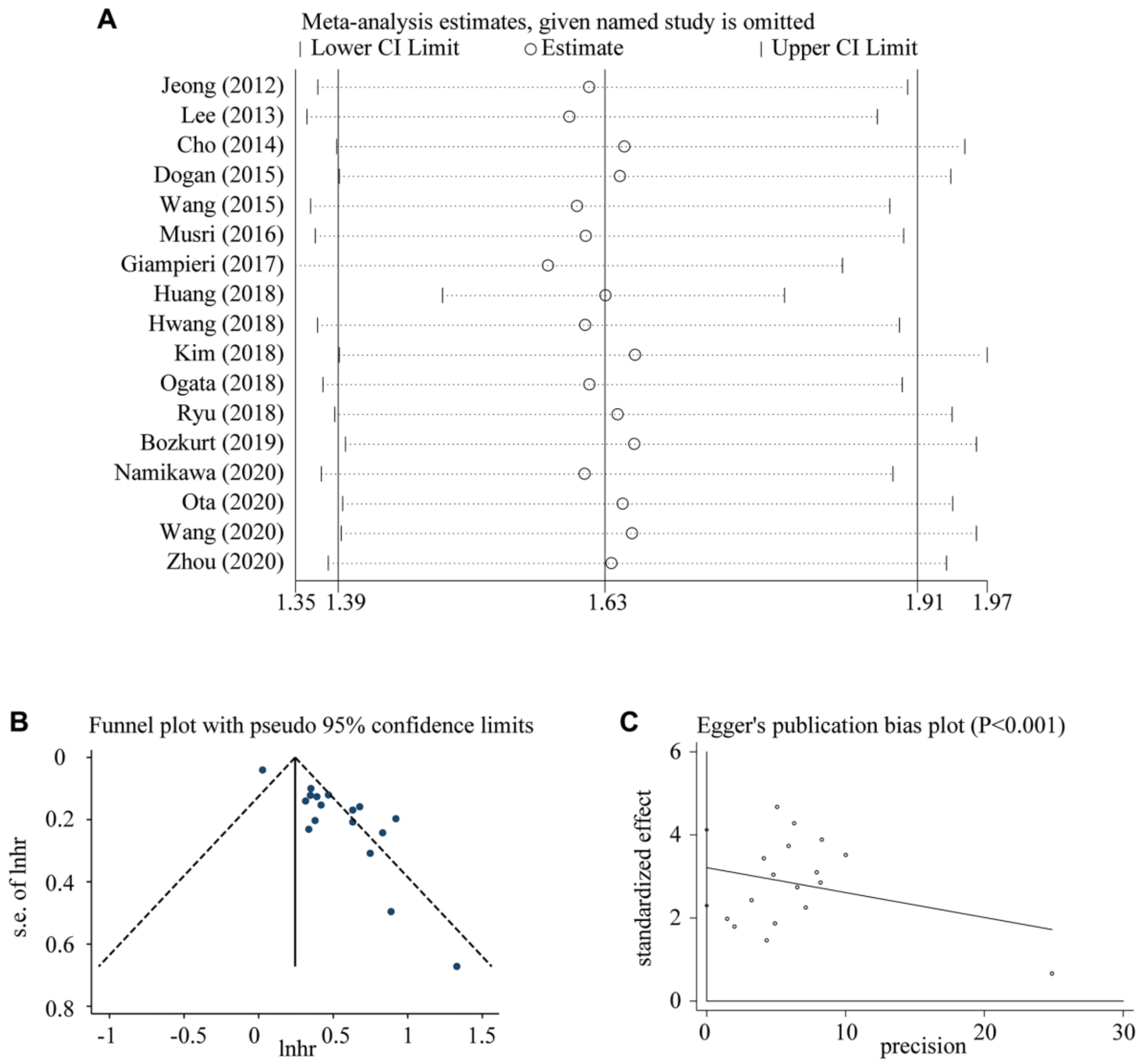

Supplementary Figure 2. Sensitivity analysis (**A**), Funnel plot (**B**) and Egger test (**C**) for the pooled HRs of PFS in gastric cancer patients with systemic therapy between low and high pretreatment NLR.
